# Synergistic Enhancement of 5-Fluorouracil Chemotherapeutic Efficacy by Taurine in Colon Cancer Rat Model

**DOI:** 10.3390/nu16183047

**Published:** 2024-09-10

**Authors:** Daniela Hartmann Jornada, Diogo Boreski, Diego Eidy Chiba, Denise Ligeiro, Marcus Alexandre Mendes Luz, Edmo Atique Gabriel, Cauê Benito Scarim, Cleverton Roberto de Andrade, Chung Man Chin

**Affiliations:** 1 Laboratory for Drug Design (LAPDESF), Drugs and Medicines Department, School of Pharmaceutical Sciences, University of São Paulo State, UNESP, Araraquara 14800-903, SP, Brazil; daniela.hj@hotmail.com (D.H.J.); diogo.boreski@unesp.br (D.B.); diego.chiba@unesp.br (D.E.C.); caue.scarim@unesp.br (C.B.S.); 2 Physiology and Pathology Department, School of Dentistry, University of São Paulo State, UNESP, Araraquara 14801-385, SP, Brazil; denise_ligeiro@hotmail.com (D.L.); cleverton.andrade@unesp.br (C.R.d.A.); 3 Advanced Research Center in Medicine (CEPAM), School of Medicine, Union of the Colleges of the Great Lakes (UNILAGO), São José do Rio Preto 15030-070, SP, Brazil; 30397@unilago.edu.br (M.A.M.L.); edag@uol.com.br (E.A.G.)

**Keywords:** taurine, colon cancer, 5-fluorouracil, chemotherapy

## Abstract

Colorectal cancer (CRC) is one of the top 10 most common cancers worldwide and caused approximately 10 million deaths in 2022. CRC mortality has increased by 10% since 2020 and 52.000 deaths will occur in 2024, highlighting the limitations of current treatments due to ineffectiveness, toxicity, or non-adherence. The widely used chemotherapeutic agent, 5-fluorouracil (5-FU), is associated with several adverse effects, including renal, cardiac, and hepatic toxicity; mucositis; and resistance. Taurine (TAU), an essential β-amino acid with potent antioxidant, antimutagenic, and anti-inflammatory properties, has demonstrated protective effects against tissue toxicity from chemotherapeutic agents like doxorubicin and cisplatin. Taurine deficiency is linked to aging and cancers such as breast and colon cancer. This study hypothesized that TAU may mitigate the adverse effects of 5-fluorouracil (5-FU). Carcinogenesis was chemically induced in rats using 1,2-dimethylhydrazine (DMH). Following five months of cancer progression, taurine (100 mg/kg) was administered orally for 8 days, and colon tissues were analyzed. The results showed 80% of adenocarcinoma (AC) in DMH-induced control animals. Notably, the efficacy of 5-FU showed 70% AC and TAU 50% while, in the 5-FU + TAU group, no adenocarcinoma was observed. No differences were observed in the inflammatory infiltrate or the expression of genes such as K-ras, p53, and Ki-67 among the cancer-induced groups whereas APC/β-catenin expression was increased in the 5FU + TAU-treated group. The mitotic index and dysplasia were increased in the induced 5-FU group and when associated with TAU, the levels returned to normal. These data suggest that 5-FU exhibits a synergic anticancer effect when combined with taurine.

## 1. Introduction

Colorectal cancer (CRC) stands as an alarming global health challenge, ranking among the most prevalent deadly cancers worldwide. According to the World Health Organization (WHO), CRC is the third most diagnosed cancer globally, with an estimated 1.9 million new cases and 935,000 deaths in 2020 and approximately 152,000 new cases in 2024 [1,2,3]. Despite advancements in early detection and treatment methodologies, CRC continues to exact a heavy toll on public health systems and individual well-being. CRC emerges through a process with various facets known as the adenoma–carcinoma sequence wherein normal colonic epithelial cells progressively transform through stages of dysplasia into invasive carcinoma. The pivotal mechanism in this progression comprises genetic and epigenetic alterations factors, including mutations in key genes such as APC, K-ras, TP53, and PTEN, alongside epigenetic modifications like DNA methylation and histone alterations, besides LncRNA [4,5,6,7,8]. Environmental factors, such as dietary habits, obesity, smoking, and chronic inflammation, further exacerbate the risk of CRC development [9].

Taurine (2-aminoethanesulfonic acid, TAU) is a major free amino acid in humans, constituting about 0.1% of body weight. It is highly concentrated in excitable tissues such as skeletal muscle, cardiac tissue, the retina, and the central nervous system (CNS [10,11,12]). While adults can synthesize taurine in the liver and CNS, newborns rely on dietary intake, classifying TAU as a semi-essential amino acid [13,14], and it is not involved in protein synthesis.

**Figure 1:**
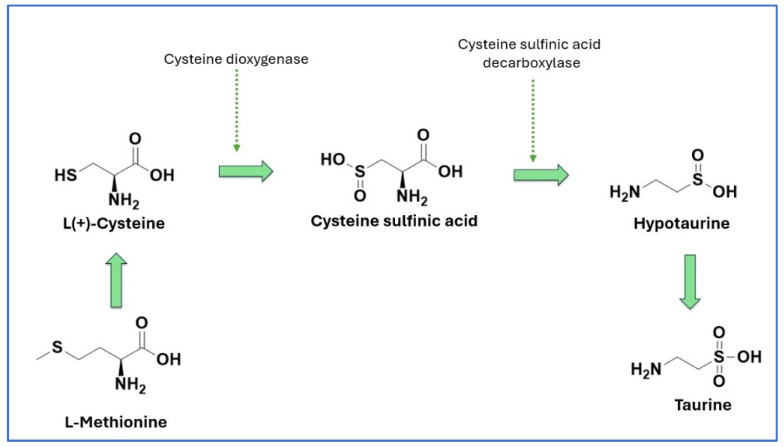
Biosynthesis of taurine.

Taurine biosynthesis primarily occurs in the liver via the cysteine sulfinic acid pathway, which involves the oxidation of cysteine into cysteine sulfinic acid, decarboxylation to hypotaurine, and subsequent oxidation to taurine [15] (Figure 1).

This process is influenced by factors such as nutritional status, protein intake, and the availability of cysteine, which depends on the balance between homocysteine and methionine, as well as the presence of folic acid and vitamin B12 [13,16].

Despite endogenous synthesis, dietary taurine remains crucial, particularly for carnivores and, to a lesser extent, omnivores [13]. Taurine homeostasis in mammals is maintained through a combination of synthesis, dietary absorption, renal reabsorption, and excretion in bile and urine [14] levels can decline by up to 80% with age, and this deficiency is associated with numerous diseases and the aging process [17]

El-Hakim et al. [18] investigated serum taurine levels as a predictive biomarker for colorectal carcinoma among Egyptian patients and compared levels with specific biomarkers before and after surgery. Some tumor biomarkers, such as Carcinoembryonic Antigen (CEA) and Carbohydrate Antigen 19.9 (CA19.9), were analyzed. While CEA and CA19.9 showed significant differences compared to controls, they remained within normal ranges for some groups. However, serum taurine levels displayed significant variations across all groups. In CRC patients, taurine levels dropped by approximately 77.5% compared to controls. In benign and inflammatory groups were observed decreases of approximately 61% and 50%, respectively, suggesting serum taurine has potential as an early biomarker for detecting malignant changes leading to CRC. The regular monitoring of taurine levels alongside other tumor biomarkers could represent a valuable method for detecting CRC, especially for individuals with gastrointestinal issues and pre-cancerous patients.

Wang et al. [19] also observed a significant reduction in tumor formation induced by AOM/DSS (toxic compounds used in cancer induction). Moreover, their study demonstrated a decrease in cellular proliferation triggered by PTEN activation, a gene responsible for suppressing the PI3K pathway and tumor growth. Taurine treatment in animals led to a decline in Ki-67 expression, indicative of reduced cell proliferation. These findings underscored the potential of taurine as a therapeutic agent in inhibiting tumorigenesis and regulating key pathways involved in cancer progression and decreasing when taurine is associated.

In a previous study [20], taurine was able to protect around 59% of the initiation of CRC in a chemically (1,2-dimethylhydrazine (DMH)) induced cancer model. Thus, as a continuation of previous research, this study aimed to investigate the effect of taurine on the progression of cancer induced by DMH in animals treated with 5-fluorouracil (5-FU). For this study, immunohistochemical analyses were performed, targeting APC/β-catenin (signaling protein), ki-67, (proliferation signaling protein) p53 (oncogene), and K-ras (proto-oncogene) for colon cancer.

## 2. Materials and Methods

The experiment was conducted according to CONCEA (National Council for Animal Experimentation) Normative Resolution 27/15 and approved by the Ethics Committee on Animal Use (CEUA) of School of Pharmaceutical Sciences (UNESP), Araraquara under protocol 07/2017.

### 2.1. Experimental Protocol

Four-week-old male Wistar rats, obtained from the Central Animal Facility at the Unesp Campus—Botucatu, were weighed and randomized divided into two groups: induced (Group A, with 51 animals) and non-induced (Group B, with 40 animals). The numbers of animals were calculate based on [20]. Group A animals received subcutaneous injections of 1,2-dimethylhydrazine (DMH) (40 mg/kg in saline, pH 6.5) twice a week for two weeks while Group B animals received saline solution (vehicle) subcutaneously, as outlined in the experimental design. The exclusion criteria included animals that died after the DMH injection but before the treatments began. There were 8 losses in the induced group, resulting in an experimental design with 43 induced animals, as described below. The induced groups were randomized into four groups, with fewer animals selected for the control group. The experimental protocol scheme is shown in Table 1.

**Figure 2:**
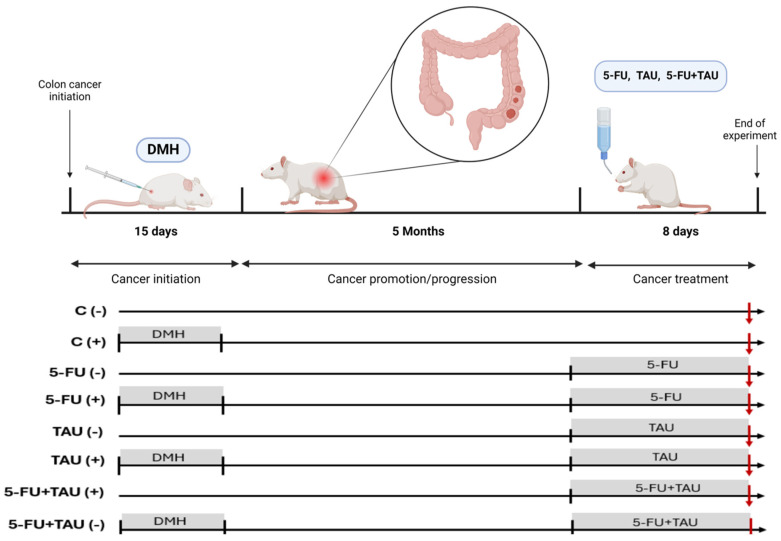
Experimental protocol design.

Throughout the experiment, the animals received autoclaved and dried food pellets and filtered water. At the beginning of the 20th week, the animals received treatments. Histopathological and immunohistochemical analyses were conducted in a double-blind experiment. On the 8th day of treatment, the animals were euthanized in a CO_2_ chamber. After the euthanasia procedure, the colon of each animal was collected and gently washed with saline solution and weighed on an analytical balance. The animals’ colons were then longitudinally opened and fixed with pins on polystyrene plates (Figure 2).

### 2.2. Colon Analysis and Processing

**Figure 3:**
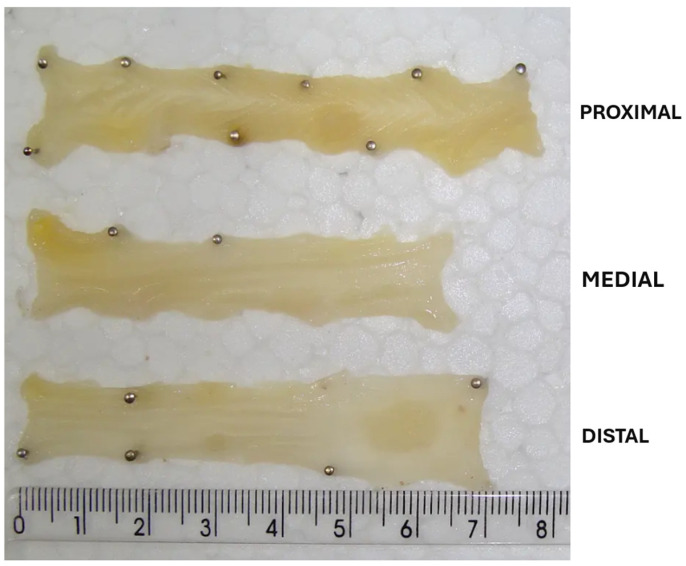
Proximal, medial, and distal portion of the colon.

Each colon was removed, rinsed with saline, measured using a ruler, and divided into three portions, proximal, medial, and distal, considering their proximity to the small intestine (Figure 3). The relative weight of each colon was not measured as the fecal material in the inner portion of the organ would be weighed collectively, which was not a relevant fraction for this study.

### 2.3. Immunohistochemical Assay

Sections of 4 μm thickness were utilized for the immunohistochemical reactions. H&E-stained sections, both initial and final, served as the foundation for subsequent immunohistochemical analyses. Negative controls were established by omitting the primary antibody in the reaction. Positive controls were obtained from samples of tongue with squamous cell carcinoma in rats induced with 4NQO (4-nitroquinoline). APC/APC/β-catenin (CAT5H10, monoclonal, IgG1, kappa, 1:1000, Invitrogen^®^, Invitriteb Thermo Fisher, Waltham, MA, USA), K-ras (9.13, monoclonal, IgG1, kappa, 1:1000, Invitrogen^®^), p53 (PAb 240, monoclonal, IgG1, kappa, 1:1000, Invitrogen^®^), and Ki-67 (SP6, recombinant monoclonal, IgG, 1:200, Invitrogen^®^) were examined.

Positive sections were identified and the original pieces were used to produce 4 μm thick sections to serve as positive controls in each series of immunohistochemical reactions containing 10 (ten) intestinal sections. Immunohistochemistry was performed using colon samples presenting neoplasia. To this end, after histological analysis of neoplasia cases, paraffin-embedded blocks were re-sectioned using a microtome and the sections were deposited on silanized slides for immunohistochemical reaction. Immunohistochemical reactions were conducted in groups of ten slides plus controls, initially kept in an oven at 60 °C for two hours to melt excess paraffin.

Subsequently, deparaffinization was carried out in four baths of 100% xylene, the first lasting five minutes and the subsequent ones two minutes each, followed by xylene/alcohol and four baths in absolute alcohol, each lasting two minutes. Gradually, water was added for rehydration (increasing concentrations), also in two-minute baths until complete rehydration was achieved. Next, antigen retrieval was performed using a 10 mM citrate buffer, with a pH of 6.2, in moist heat, at 95 °C in a pressure cooker. Once reconstituted, endogenous peroxidase was inactivated by exposing the tissues to hydrogen peroxide for ten minutes at room temperature.

Finally, nonspecific sites were blocked with blocking solutions for ten minutes. Each antibody used strictly followed the specifications described in its datasheet. Concentrations suggested by the manufacturers were considered as the standard (gold) reaction, and overnight incubation in a refrigerator was conducted to enhance specificity by reducing molecular motility. For this purpose, a hermetic and humidified dark chamber was used, allowing the slides to remain flat under adequate humidity until the end of the reaction time.

The primary antibodies used were APC/β-catenin (CAT-5H10, monoclonal, IgG1, kappa, 1:1000, Invitrogen^®^), aiming to verify the presence of cell-proliferation-related mutations characteristic of the promotion process; K-ras (9.13, monoclonal, IgG1, kappa, 1:1000, Invitrogen^®^), aiming to verify the presence of mutations in later stages of the promotion process; p53 (PAb 240, monoclonal, IgG1, kappa, 1:1000, Invitrogen^®^), serving as an indicator of non-responsiveness to apoptotic stimuli and malignancy; and Ki-67 (SP6, recombinant monoclonal, IgG, 1:200, Invitrogen^®^), serving as a marker of proliferative activity. A hydrophobic pen was also used to encircle the sections to prevent runoff. Incubation with the secondary antibody conjugated with peroxidase (Goat antiRabbit IgG, AP (AP, polyclonal, 1:5000, Invitrogen^®^) was conducted for thirty minutes at a concentration of 1:150, followed by reaction with the chromogen 3,3’-diaminobenzidine tetrahydrochloride (DAB), and counterstained with Meyer’s Hematoxylin.

Finally, the histological sections were dehydrated in increasing alcohol concentrations, followed by clearing in xylene baths, each lasting two minutes. The antibodies were diluted in a 1% bovine serum albumin solution (Sigma-Aldrich^®^, St. Louis, MO, USA) in 1X PBS. Each marker was standardized using the manufacturer’s standard concentration, as the initial parameter, in addition to concentrations 10 times lower and 100 times lower. After verifying labeling, the times were noted and used as standards for subsequent reactions using the same secondary antibody. The identification of background presence or adjustments to chromogen exposure time was noted and strictly followed in each immunohistochemical reaction of each marker.

Immunohistochemical analysis was performed on the neoplastic lesions observed in the histological analysis. The analysis of the immunohistochemical expression was carried out using two parameters: (Q) the quantity (<10% (1), 11–25% (2), 26–50% (3), >51% (4)) and (I) the intensity (none (0), low (1), moderate (2) or high (3)). The score of the labeling (S) was obtained by using the mathematical formula S = Q X I; score could be low (0–4), moderate (5–8), or high (9–12) [24].

### 2.4. Statistical Analysis

When data met the assumption of normality, Analysis of Variance (ANOVA) was employed, and the post-test conducted to identify differences between groups was Bonferroni test. Data that did not meet the assumption of normality (Shapiro–Wilk) were analyzed using the Kruskal–Wallis test, followed by the Dunn test. The significance level used for decision making was 5% (*p* < 0.05).

## 3. Results

**Figure 4:**
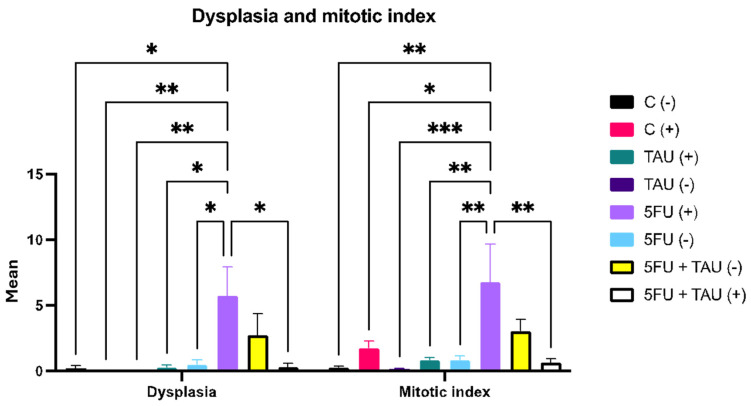
Dysplasia and mitotic index in animals induced with DMH. (-) group without DMH induction; (+) group with DMH induction; * *p* < 0.5; ** *p* < 0.01; *** *p* < 0.001.

The results of dysplasia and the mitotic index showed a notable difference between the 5FU (+) group and the other groups (Figure 4). Dysplasia can be understood through the drug’s mechanism of action, which interferes with DNA and RNA synthesis. Histologically, this interference can cause tissue dysplasia, although the primary objective is to eliminate colon cancer.

**Figure 5:**
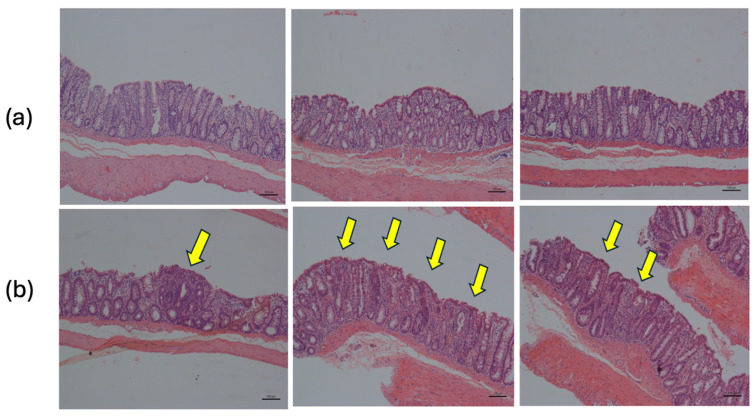
Histology of distal colon tissue. (**a**) Normal tissue: simple columnar epithelium with thin brush border and numerous goblet cells. (**b**) Dysplastic tissue: regions of dysplastic epithelium are indicated by arrows (H&E 10X).

It is evident that 5FU contributes to promoting dysplasia as the combination group showed a difference compared to the other experimental groups. This effect was observed in the non-induced groups that received 5-FU as treatment, either individually or in combination. This may be related to the fact that they did not undergo the initiation process (chemical induction), which would predispose them to the appearance of these lesions, making 5FU the incremental factor. Figure 5 shows a normal colon and dysplasia of the colon.

Regarding the mitotic index data, a similar effect could be observed. The groups showing an increased mitotic index were those that had been induced and treated with 5FU. The induction factor seemed more predominant in this case as there was an increase in the mitotic index in the C (+) group. This aligned with the literature as the mitotic index measures the rate at which tissue cells are dividing, which is indicative of the likelihood of tumor area dissemination. Thus, after animals have undergone carcinogen induction, an increase in mitotic indices is expected, leading to the promotion and subsequent progression phases. Although the initiation stage occurred 20 weeks prior to treatment, it was not possible to predict the specific phase each animal was in or the specific phase of each part of the colon as animals with more than one neoplastic lesion were also observed in the experiment.

**Figure 6:**
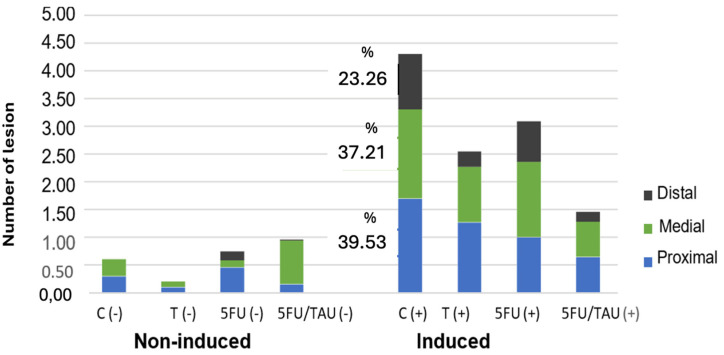
Distribution of the visible lesions of the colon in animals induced with DMH.

Among the induced groups, there was a difference only in the total count of lesions. It was observed that TAU (+), 5FU (+), and 5FU + TAU (+) presented fewer lesions than the control C (+), with significant values of *p* < 0.01, *p* < 0.05, and *p* < 0.001, respectively. Additionally, TAU (+) and 5FU (+) were equivalent to each other, but when comparing TAU (+) with the combination group (5FU + TAU (+)), there was no statistical difference between the groups. However, for 5FU (+) and 5FU + TAU (+), it was observed that the combination group promoted a greater reduction in the total number of visible lesions than the standard treatment (*p* < 0.01), demonstrating that TAU improves the activity profile of 5FU in reducing lesions. Figure 6 shows the distribution of these lesions in the colon (proximal, medial, distal).

Table 2 shows the results of the histological parameters analyzed. Regarding the characteristics of submucosal layer inflammation, 100% of the animals exhibited mixed-origin inflammation, with the presence of neutrophils, macrophages, and lymphocytes, while the intensity ranged between mild and moderate levels. We can observe the improvement of the parameters with the treatment of 5FU associated with TAU in the induced groups compared to 5FU (+) alone.

**Figure 7:**
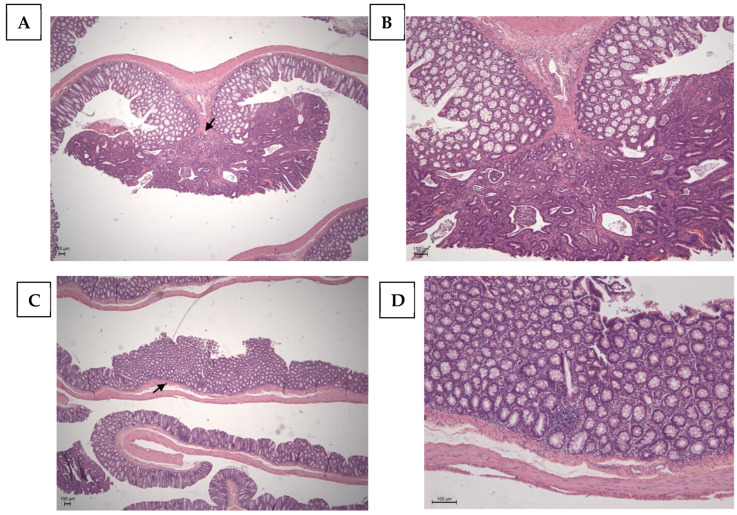
Histopathological image of the positive control with chemical DMH induction. (**A**,**B**): Adenocarcinoma of the colon; the arrow points to the invasion area. (**C**,**D**): Adenoma of the colon; the arrow points the absence of invasion. Calibration bar: 100 µm.

Table 3 shows the percentage of neoplasia found by each group. In the C (+) group, adenomas represented 20% of the lesions while adenocarcinomas accounted for 80%. In the TAU (+) group, adenomas and adenocarcinomas each represented half of the lesions. The group treated with 5FU (+) showed 70% adenocarcinomas and 30% adenomas. However, when associated with taurine (5FU + TAU (+)), no adenocarcinomas were observed, only adenomas. The images of lesions observed in the histopathological analysis of the colon are presented in Figure 7.

### Immunohistochemistry

Immunohistochemical analyses were performed on the neoplastic lesions observed in the histological analyses only for the positive slides (DMH-induced). The control group, C (+), presented twenty (20) neoplastic lesions; the TAU (+) group presented thirteen (13) neoplastic lesions; the 5FU (+) group presented twenty (20) neoplastic lesions; and the 5FU+TAU group presented twelve (12) neoplastic lesions. The results of the immunohistochemical analyses for β-catenin, p53, K-ras, and Ki-67 showed that the TAU (+), 5FU (+), and 5FU+TAU (+) groups had higher β-catenin scores (Table 4).

**Figure 8:**
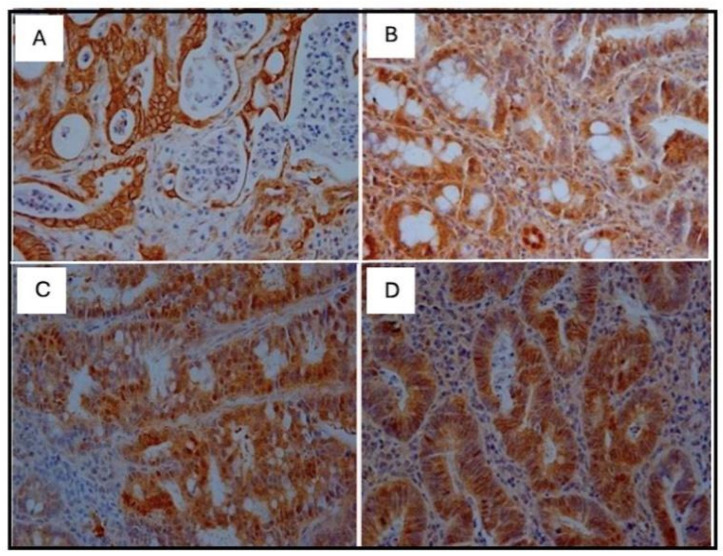
Immunohistochemical images of β-catenin-reactive cells. (**A**): C (+); (**B**): TAU (+); (**C**): 5FU (+); (**D**): 5FU + TAU (+) (5× magnification).

**Figure 9:**
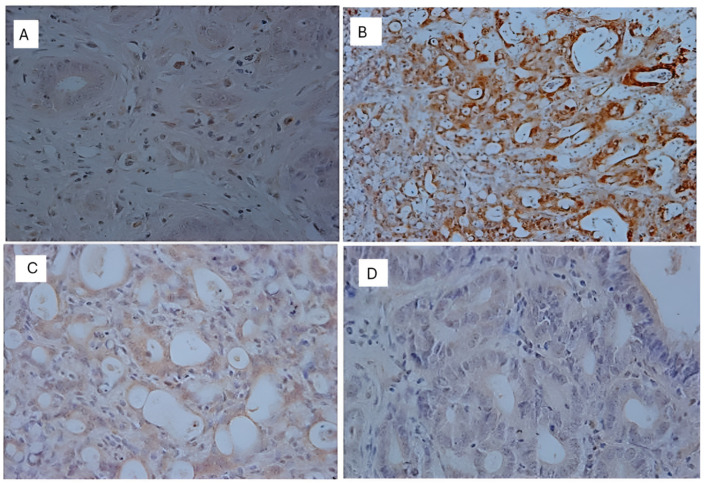
Immunohistochemical images of p53-reactive cells. (**A**): C (+); (**B**): TAU (+); (**C**): 5FU (+); (**D**): 5FU + TAU (+) (5× magnification).

**Figure 10:**
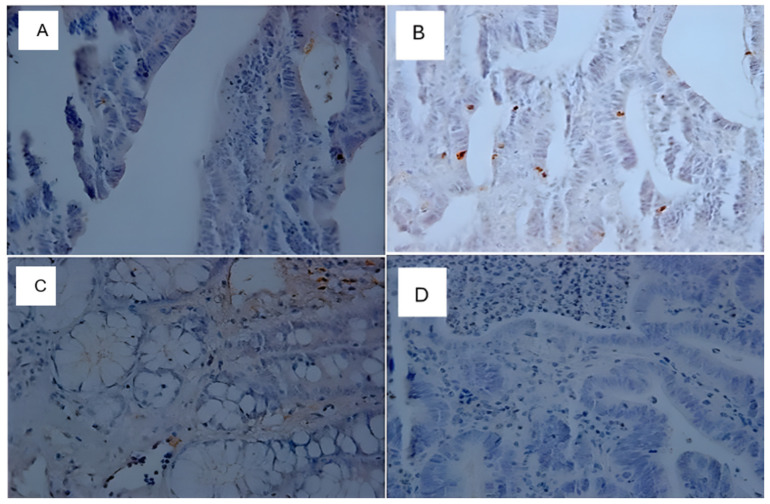
Immunohistochemical image of Ki-67-reactive cells. (**A**): C (+); (**B**): TAU (+); (**C**): 5FU (+); (**D**): 5FU + TAU (+) (5× magnification).

**Figure 11:**
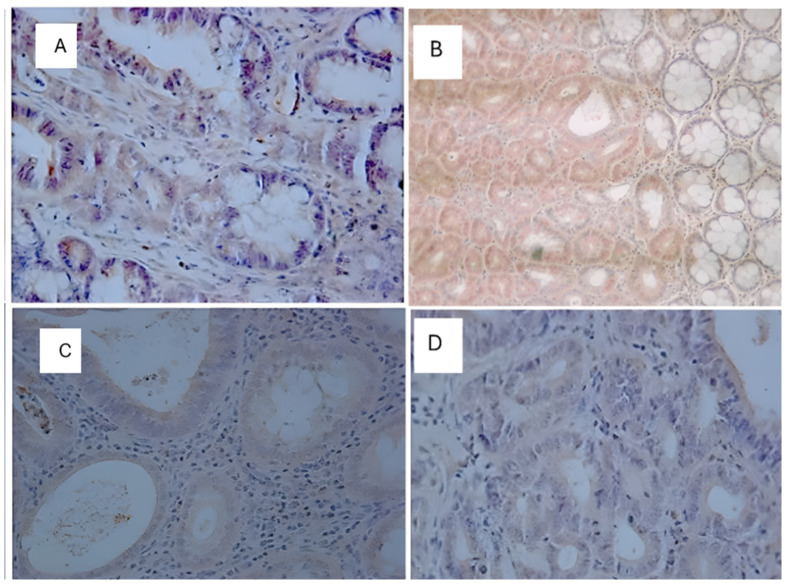
Immunohistochemical image of K-ras-reactive cells. (**A**): C (+); (**B**): TAU (+); (**C**): 5FU (+); (**D**): 5FU + TAU (+) (5× magnification).

When analyzing β-catenin, the C (+) group had a lower score compared to the 5FU (+) and 5 FU+TAU (+) groups, and the TAU (+) group had a lower score than the 5FU (+) and 5FU+TAU (+) groups, while the 5FU+ group had a higher score than all the groups studied. On the other hand, when we analyzed the K-ras staining, we observed that the 5FU+TAU (+) group had a higher score than the TAU (+) and C (+) groups. Figure 8, Figure 9, Figure 10 and Figure 11 show the images of the immunohistochemical reactions for β- catenin, p53, Ki-67, and K-ras.

## 4. Discussion

Several new studies involving taurine and cancer have been reported recently [21,25,26,27]. The biological activity of taurine against various types of tumors, such as breast, liver, Ehrlich-ascite, and colon cancers, has gained prominence, both for its chemopreventive effect on carcinogens and for its apparent inhibitory effect on tumor cell proliferation, whether used alone or in combination. Studies with derivatives of conjugated forms of taurine, such as tauroursodeoxycholic acid, have shown its ability of attenuating colon cancer by inhibiting the nuclear factor kappa B (NF-kB) pathway [28]. Furthermore, in our previous study, the in vivo antimutagenic effect of taurine promoted by mutagenic drugs (such as nitro compounds) was shown to range between 45 and 79%, depending on the substance, by blocking taurine from chromosomes, interfering with cell division, or by its antioxidant effect against nitro bioreduction by-products [29]. Our findings corroborate the results of Wang and colleagues and suggest that taurine may act in the chemoprotection of early colon carcinogenesis by reducing the multiplicity of aberrant crypt foci, which are considered biomarkers in the progression of colorectal cancer [30].

Interestingly, our results from taurine treatment and the induction of cancer with DMH suggest a specific activity in the proximal colon, which may be related to taurine’s solubility and permeation in the colon. Taurine is highly soluble in water at the physiological pH. However, as it is in its ionized form, transmembrane proteins are required for taurine to be taken up into the cell (ion-dependent transport of Na+ and Cl- or H+) [31]. The main proteins are the TauT receptors (Taurine Transporter), high-affinity transporter with very low capacity, and PAT1 (Proton-coupled amino acid transporter-1), a low-affinity but high-capacity transporter capable of capturing circulating amino acids. The intraluminal transport of taurine in the kidney is regulated according to the demand for nutrients; in this case, the occurrence of taurine in its soluble form is a determinant for the exposure of its specific transporter [32,33]. Studies on the intestinal taurine transporter have shown that PAT1 expression is more abundant in the jejunum, about 1.5 times higher than in other parts of the intestine, and TauT seems to be distributed similarly, although expression in the proximal colon is especially lower than in other regions [32,34]. This may suggest that the lower concentration of taurine transporters in the proximal portion allows the amino acid to remain in the intestinal lumen longer, exerting local activity [35].

The results obtained with taurine combined with the chemotherapeutic drug 5-FU after induction showed that the average number of tumors in the induced group treated with 5-FU was approximately 28% higher than the average for the positive control. The amino acid showed approximately 64% fewer neoplasms than the positive control and approximately 72% fewer than the standard drug, 5-FU (+), in the induced group receiving taurine alone.

As regards the combination, the incidence of neoplasms was approximately 82% lower than in the C (+) group, 86% lower than in the 5-FU (+) group, and 50% lower than in the group treated with taurine alone (TAU (+)). It is important to note that the neoplasia data in the 5-FU (+) group do not indicate that the drug causes neoplasia. The count being higher than the positive control was due to an incremental factor of the carcinogenesis promotion process in the animal model; since 5-FU is an uracil analogue, it can cause an incremental mitosis in health cells. Indeed, DMH is considered a complete carcinogen, requiring no other substances to promote carcinogenesis. However, because of its mechanism of action, 5-FU may have acted to increase tumorigenesis, justifying a score higher than C (+). In addition, the drug did not lead to the development of tumors in the non-induced group that received 5-FU. Moreover, there was a synergistic activity of taurine under this point as a reduction in the number of neoplasms was observed in the group that received the combination, and this reduction was higher than the value obtained for isolated taurine.

Our findings are consistent with the existing literature, exemplified by Zhang et al. and Wang et al. [19,21], who demonstrated taurine’s ability to promote a 40–50% reduction in colon tumors in xenograft models employing LoVo and HT29 cell lines. Taurine induced apoptosis in tumor cells and inhibited their proliferation. Molecular mechanisms involved the upregulation of the PUMA gene, increased expression of the proapoptotic gene Bax, and downregulation of the anti-apoptotic gene Bcl-2, thereby activating caspase 3 and 9. However, the sole reliance on these mechanisms is contested, as evidenced by the observation that p53 gene deletion did not impede taurine-induced apoptosis.

Taurine’s tumor reduction may be closely related to its ability to alter the intracellular levels of inflammatory cytokines. Serum concentrations of tumor necrosis factor alpha (TNF-α), interleukin 17 alpha (IL-17α), regulated-upon-activation normal T-cell expressed and presumably secreted (RANTES), interleukin 1 alpha (IL-1α), and granulocyte–macrophage colony-stimulating factor (GM-CSF) were found to be elevated in middle-aged mice compared to young mice. Similarly, taurine showed the ability to modulate epigenetics such as histone methylation and chromatin, suggesting taurine may affect chromatin conformation as observed by Singh et al. [17]. Studies by Liu et al. [25] further supported these findings, showing that taurine-induced apoptosis in colorectal cancer cells (Caco-2 and W620) occurs via the MST1-JNK pathway, albeit with reduced efficacy in MST-1 gene deletion scenarios. They proposed that taurine’s apoptotic action may involve three signaling pathways: MST1-JNK, MST1-Hippo (associated with the p53 pathway), and PUMA (mitochondrial apoptosis pathway).

No statistical variance was found between the groups analyzed regarding the quantification of the inflammatory infiltrate. Nevertheless, with respect to submucosal inflammatory characteristics, all animals displayed mixed-origin inflammation, characterized by the presence of neutrophils, macrophages, and lymphocytes, with intensity ranging from mild to moderate. As no reduction in local inflammatory processes was observed, this suggests that the effects of taurine may be due to other activities rather than direct anti-inflammatory effects.

Notably, significant differences were observed between the 5-FU (+) group and other groups regarding dysplasia and the mitotic index. Dysplasia, synonymous with intraepithelial neoplasia, can be elucidated through the pharmacological mechanism, which disrupts DNA and RNA synthesis, histologically resulting in tissue dysplasia, despite the primary objective being colon cancer elimination. Chemical carcinogenesis in our experiment facilitated colon tumor formation in a nonspecific manner. Thus, even in the case of animals with pre-established tumors, dysplastic formations may arise due to the drug’s action at other colon sites. As shown by the deviation of the combination group from the other experimental groups, the contribution of 5-FU to dysplasia was obvious. However, this phenomenon was not observed in non-induced groups receiving 5-FU treatment, whether individually or in combination. This could be attributed to the absence of an initiation phase (chemical induction), which serves as a predisposing factor for lesion appearance, making 5-FU an incremental factor, as previously discussed for neoplasia. Similarly, an increase in the mitotic index was noted in the groups, particularly those induced and treated with 5-FU.

The inducer appears to have a greater influence here, as shown by an increase in the mitotic index in group C (+). This finding is in line with the existing literature as the mitotic index serves as a measure of the rate of cell division in tissues and, in the case of tumors, of the likelihood of their spread. Thus, after induction with the carcinogen, increased mitotic indices would be expected to facilitate promotion and progression. Although the induction phase occurred 20 weeks prior to treatment, it remains difficult to determine the specific phase of each animal or even the specific phase of each colon segment. This is because animals with multiple neoplastic lesions were observed in the experiment. Furthermore, the combined group showed a reduction in both dysplasia and the mitotic index, highlighting the reducing effect associated with taurine administration.

The immunohistochemical analysis of the lesions revealed an interesting finding: a significant increase in the β-catenin marker in the 5FU + TAU group despite fewer lesions and fewer animals with adenocarcinoma being observed in histology. 

APC mutations are hypothesized to be the initial events in sporadic colon adenomas. The primary function of APC is the regulation of free β-catenin in conjunction with glycogen synthase kinase 3β (GSK-3β) and other proteins [36]. It has been discovered that half of all human colon tumors with an intact APC protein have a mutation in the β-catenin gene [37]. APC mutations have also been identified in colorectal cancer and epithelial lesions in the DMH/AOM rat model, although to a lesser extent and in a different region than that observed in humans [38]. In DMH-treated rats, up to 33% of colon tumors harbor APC mutations. In the DMH rat model, β-catenin mutations are more frequent than APC mutations, occurring in up to 77% of tumors. These are primarily point mutations (G to A transitions) located in the GSK-3β phosphorylation consensus motif, resulting in the inhibition of GSK3-dependent β-catenin phosphorylation.

However, in normal epithelial cells, β-catenin is highly expressed in the cell membrane and not detected in the plasma or cell nucleus [39]. It has been observed, in dysplastic colorectal epithelial lesions (including adenomas and adenocarcinomas), but not in hyperplastic lesions, that β-catenin expression is increased in the cytosol and nucleus [40,41].

The K-ras gene encodes a membrane-bound protein with intrinsic GTPase activity in the colon that is involved in regulating a series of important normal cellular functions, including proliferation, differentiation, and apoptosis. Single-point mutations at specific sites within ras genes activate their oncogenic potential. K-ras mutations have been observed with varying frequencies (~40–50%) in human colorectal neoplastic lesions, as well as in ACF with a hyperplastic epithelium. It has been reported that K-ras is mutated in fewer than 10% of small adenomas (less than 1 cm in size), in about 50% of large adenomas (greater than 1 cm), and in approximately 50% of carcinomas. However, some studies have shown that K-ras mutations are less frequent in small adenomas (average ~16%) and more frequent in large adenocarcinomas (~53%) [42]. Based on these studies, it appears that K-ras mutations in the DMH/AOM rat model are as frequent as in human colon carcinogenesis. As in humans, a K-ras mutation was more frequently observed in ACF with a hyperplastic epithelium than in ACF with dysplastic characteristics [43]. The K-ras mutation may, therefore, be involved in ACF lesion formation and growth promotion, but it is probably not essential.

Our research results showed no changes in the p53 gene. Although p53 gene aberration is a very common genetic lesion identified in human carcinomas, these changes were not found in DMH-induced rat carcinogenesis [44]. The proliferation antigen Ki-67 is expressed in all phases of the cell cycle and is reliable as a proliferative marker, being strictly associated with cell proliferation. The Ki-67 protein is present during all active phases of the cell cycle (G1, S, G2, and mitosis) and absent in quiescent cells (G0) [45]. Our results showed low staining even using different batches of Ki-67 and did not demonstrate statistical differences between the cancer-induced groups, although we observed differences in mitotic indices. The mitotic index quantifies mitoses in ten 400X fields. These results, analyzed together, demonstrated that the cell cycle was not altered when we verified Ki-67 expression, but also that there was possibly a dragging of cells into mitosis (mitotic index). Cell cycle dysregulation may be associated with the resistance of gastric cancer cells to 5-FU treatment [46].

**Figure 12:**
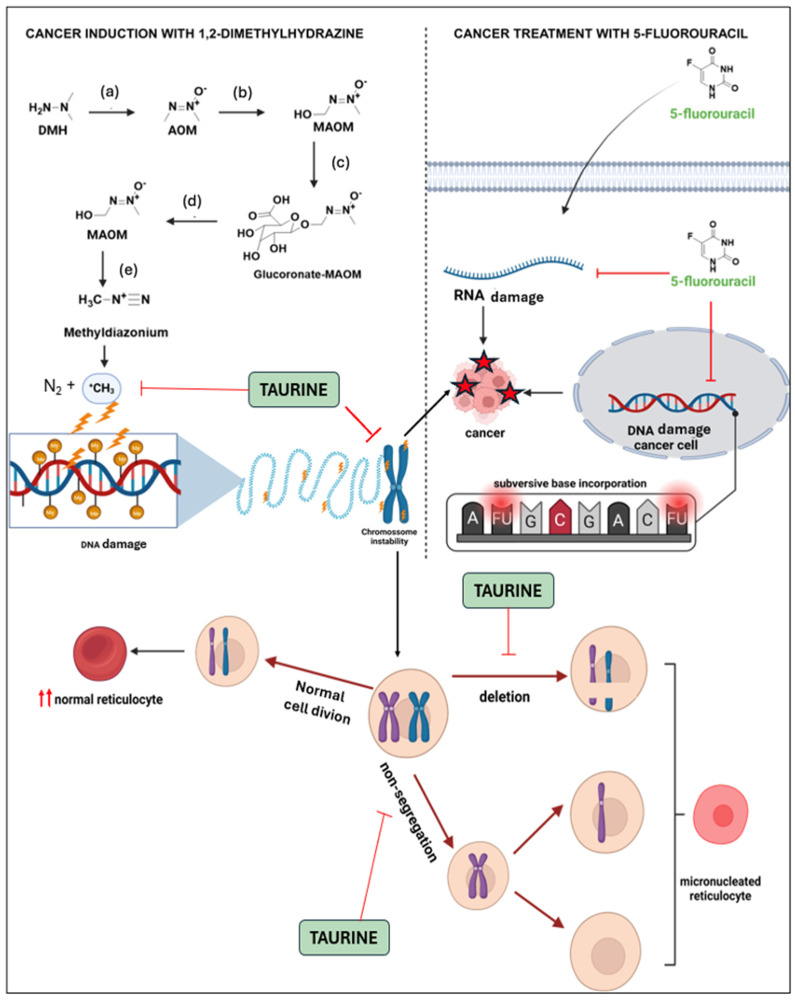
Mechanism of DMH-induced colon cancer and synergic effect of taurine and 5-FU against cancer growth. DMH is oxidized (**a**,**b**) in the liver by phase I enzymes into azoxymethane (AOM) and subsequently into methylazoxymethanol (MAOM). This compound is then glucuronidated by phase II enzymes and excreted through the bile (**c**). In the intestine, in the presence of beta-glucuronidase from colonocytes or the microbiota (**d**), MAOM is transformed back and can spontaneously convert into methyldiazonium (**e**), which is considered the ultimate carcinogen due to its ability to alkylate nitrogenous bases [47] The chromosomal instability generated by DMH can be blocked by TAU since it is able to act as a potent antioxidant, reacting with free radicals and stabilize the chromosome, observed by the decrease in the number of micronucleated reticulocytes caused by mutagenic compounds [29]. The chemotherapeutic antimetabolic agent 5-FU replaces uracil during protein synthesis, inhibiting thymidylate synthase and being mistakenly incorporated into DNA, which triggers cell death [48].

We propose a synergic mechanism of TAU’s protective effect in combination with 5 FU, based on cancer induction by DMH, which may involve two pathways: free radical formation following its metabolism and genomic instability as shown in Figure 12.

New studies have highlighted the role of long non-coding RNAs (lncRNAs) in neoplastic development and progression. LncRNAs, longer than 200 nucleotides, play critical roles in various biological processes including cell proliferation, differentiation, development, apoptosis, and metastasis [49,50]. By chance, taurine is involved in the upregulation of the expression of a specific lncRNA [51]. Taurine upregulates gene 1 (TUG1), a gene located at 22q12.2 on chromosome 22, with its highest expression observed in the retina and brain [52]. In vitro studies have indicated the involvement of TUG1 in the development of various types of cancers, such as breast cancer [53], ovarian cancer [54], and colorectal cancer [55]. However, the roles in biological processes and the mechanisms of action for the majority of lncRNAs remain undetermined.

LINC00261 levels have been reported to be reduced in colon cancer tissue, as shown by Chen et al. [56]. In this way, increasing the expression of LINC00261 has been shown to inhibit the viability of colon cancer cells by either preventing the export of β-catenin from the cytoplasm to the nucleus or promoting its degradation [22,23]. TUG1 is emerging as a potential biomarker in colorectal cancer (CRC) for early detection, prognosis prediction, and evaluating therapeutic responses. The current research suggests that TUG1 is a major contributor in CRC metastasis. High levels of TUG1 in tumor tissue were shown to correlate with poor patient survival in an analysis of TUG1 expression in 120 CRC patients [22,57,58]. In vitro studies have also shown that the upregulation of TUG1 in CRC cell lines has oncogenic effects. It increases colony formation, the migratory capacity, and metastatic potential. Increased TUG1 expression was found to further stimulate these oncogenic behaviors in a xenograft animal model. This model also showed that TUG1 switched on genes involved in EMT, a critical process in cancer metastasis. These results highlight TUG1’s role in colorectal cancer progression and its potential as a target for developing new therapeutic strategies and expand our understanding of factors involved in cancer development and progression [58].

Moreover, in a study conducted by Cao et al., [59] the TAU transporter SLC6A6 (TAUT) was linked to increased aggressiveness and poor prognosis in several cancer types. SLC6A6-facilitated TAU uptake supports the malignant behavior of tumor cells while also promoting the survival and function of CD8+ T cells since TAU is the most abundant free amino acid in the leukocyte’s cytoplasm. Tumor cells, by overexpressing SLC6A6, outcompete CD8+ T cells for TAU, leading to T cell death and dysfunction, which in turn drives tumor progression. Mechanistically, taurine depletion in CD8+ T cells exacerbates ER stress, triggering ATF4 transcription through a PERK-JAK1-STAT3 signaling pathway. This elevation in ATF4 activates various immune checkpoint genes, contributing to T cell exhaustion. In gastric cancer, the study identified a chemotherapy-induced SP1-SLC6A6-regulatory axis. These findings suggest that tumor-induced SLC6A6-mediated taurine deficiency promotes immune evasion while taurine supplementation revitalizes exhausted CD8+ T cells and enhances the effectiveness of cancer therapies, aligning with our results. In addition, the researchers investigated overexpressed genes using qPCR in blood samples from six CRC patients and found that the gene SLC6A6 was the most overexpressed gene.

Interestingly, over the last decade, studies involving the drug Taurolidine have emerged. Taurolidine was synthesized in the 1970s by Geistlich-Pharma as a broad-spectrum antibiotic, but it has been tested as a promising substance in inducing apoptosis in tumor cells. In vitro studies have determined that 20 mg/kg of Taurolidine exhibits an inhibitory effect on cellular proliferation with low toxicity in cellular models of ovarian cancer (PA-1 and SKVO-3) [60].

The initial clinical experience with taurolidine as an antineoplastic agent was documented in a study by Stendel et al., involving two male patients with histologically confirmed glioblastoma. Following taurolidine administration, both patients exhibited improved neurological conditions and quality of life. Additionally, partial tumor remission was observed in both cases [61].

The precise mechanism by which taurolidine affects tumor cells remains not fully elucidated. Evidence suggests that it promotes apoptosis, inhibits angiogenesis and tumor cell adhesion, reduces levels of proinflammatory cytokines and endotoxins, and stimulates the immune response following surgical trauma. Notably, taurolidine consistently induces apoptosis and inhibits angiogenesis across various cancer cell types, though the extent to which apoptosis contributes to cell death varies among different cancers [60,62,63]. However, it has been hypothesized that the suppression of tumor growth may be attributed to intracellular mechanisms inducing apoptosis, potentially through a mitochondrial cytochrome-C-dependent apoptotic pathway or a caspase-dependent pathway [62]. Despite data and studies indicating an antineoplastic effect of taurine and its derivatives, further studies are necessary to better understand the mechanism by which this amino acid operates and its viability as part of an adjunct clinical therapy.

The primary limitation of this study was the relatively short duration of the treatment. Initially, our goal was to investigate the integrity of colon tissue by TAU to prevent bacterial translocation during chemotherapy. This was based on preliminary unpublished studies from our laboratory that demonstrated impressive recovery from intestinal necrosis using TAU in an acetic-acid-induced colitis model. Unexpectedly, we observed a reversal from adenocarcinoma to adenoma within just 15 days in this work, and to accurately assess the complete response or normalization of biomarkers, a more extended evaluation period is required.

Our findings suggest that TAU could serve as a valuable cancer adjuvant by mitigating the adverse effects of 5-FU while potentially enhancing its anticancer efficacy through a synergistic mechanism. Given the potential for translating these results to clinical practice for CRC patients, a dosage of 100 mg/kg of TAU is safe. According to Merck, Inc. (Darmstadt, Germany) [64], the acute toxic dose of taurine is approximately 2500 mg/kg, and the LD50 in rats is greater than 2000 mg/kg. This means that the 100 mg/kg dosage is well below the toxic threshold for humans, being 20 times lower than the estimated toxic dose of approximately 140 g for a 70 kg person. Therefore, the application of a 100 mg/kg taurine treatment in CRC patients is feasible, given its low toxicity profile.

Furthermore, a 2023 study by Singh et al. involving mice, monkeys, and humans highlighted taurine’s role in influencing the aging process, increasing lifespans. Additionally, a clinical trial using a 6 g dose of taurine demonstrated its safety and effectiveness in reducing portal hypertension [65]. These findings strengthen the rationale for studying taurine’s effects in humans.

## 5. Conclusions

In conclusion, our study suggested a protective role of TAU in the initiation and progression of colon cancer chemically induced by DMH. However, the underlying mechanisms remain to be explored. In combination with the chemotherapeutic agent 5-FU, TAU significantly reduces lesion occurrence. Histopathological analyses indicated that taurine’s effect is not due to its anti-inflammatory activity. Immunohistochemical assays of positive samples (adenomas and adenocarcinomas) demonstrated a significant increase in β-catenin expression, which may have been associated with enhanced cellular normality, partially explaining the observed reduction in the number of lesions (decreased percentage of adenocarcinomas and increased percentage of adenomas). Regardless of the mechanism, we strongly recommend conducting a clinical trial to evaluate the use of TAU as an adjuvant in 5-FU treatment for colon cancer. This trial would help confirm the potential benefits of taurine in enhancing the antineoplastic efficacy of 5-FU while mitigating its deleterious and toxic effects, ultimately improving outcomes for patients with colorectal cancer.

## Figures and Tables

**Table 1:** Experimental protocol of cancer induction using 1,2-dimethylhydrazine (DMH).

Group A—Induced (DMH) (43)		Group B—Non-Induced (Saline Solution) (40)	
C (+)	Vehicle * (10)	C (-)	Vehicle * (10)
TAU (+)	Taurine ** (11)	TAU (-)	Taurine ** (10)
5-FU (+)	5-FU *** (11)	5-FU (-)	5-FU *** (10)
5-FU+TAU (+)	Taurine ** e 5-FU ***; (11)	5-FU+TAU (-)	Taurine ** e 5-FU *** (10)

(n) = number of animals. * Vehicle: Saline solution intraperitoneal injection (i.p.) and gavage water. ** Taurine: 100 mg/kg (gavage) water solution [21]. *** 5-FU: Cycle 1 comprised 4 days with 12 mg/kg (i.p.) and was followed by cycle 2: 4 days with 6 mg/kg (i.p.) in saline solution [22,23].

**Table 2:** Table of selected histological parameters analyzed for the distal colon.

	C (-)	C (+)	TAU (-)	TAU (+)	5FU (-)	5FU (+)	5FU + TAU (-)	5FU + TAU (+)	
Adenoma (A)	0.00	0.10 ± 0.10 ^a^	0.00	0.09 ± 0.09 ^a^	0.00	0.18 ± 0.12 ^a.^	0.00	0.09 ± 0.09 ^a^	
Adenocarcinoma (AC)	0.00	0.40 ± 0.22 ^a,d,h^	0.00	0.09 ± 0.09 ^a,b,f,h^	0.00	0.45 ± 0.20 ^a,d,h^	0.00	0.00 ^a,b,d,f^	
Inflammatory infiltrate	0.59 ± 0.09	0.56 ± 0.06	0.65 ± 0.15	0.67 ± 0.09	0.94 ± 0.13 ^a,c,d,f,h^	0.67 ± 0.06	0.59 ± 0.09	0.71 ± 0.08 ^e^	
Submucosal inflammation Intensity	2.25 ± 0.01	2.20 ± 0.08	2.50 ± 0.11	2.82 ± 0.10 ^g^	2.50 ± 0.13	2.27 ± 0.12	2.25 ± 0.12	2.36 ± 0.12	
Mucosa—loss of continuity	0.40 ± 0.16	0.10 ± 0.10 ^i^	0.20 ± 0.13 ^a,b,e,f,g,h^	0.18± 0.12	0.0 ^g^	0.91 ± 0.09	0.40 ± 0.16 ^b,c,d,e^	0.55 ± 0.16 ^i^	
Mucosa appearance	1.80 ± 0.25	2.00 ± 0.15	1.80 ± 0.13	2.00 ± 0.19	2.10 ± 0.10	1.09 ± 0.09 ^i^	1.80 ± 0.25	1.36 ± 0.15 ^i^	
Muscular—loss of continuity	0.00	0.00	0.00	0.00	0.00	0.00	0.00	0.09 ± 0.09	
Muscular tissue appearance	1.6 ± 0.16	1.90 ± 0.10	1.80 ± 0.13	1.82 ± 0.18	2.10 ± 0.10	1.18 ± 0.12 ^b,c,d,e,g^	1.60 ± 0.16	1.36 ± 0.15 ^b,c,d,e,g^	
Mucus production	2.20 ± 0.24 ^h^	2.05 ± 0.09 ^h^	1.95 ± 0.14 ^h^	1.95 ± 0.20 ^h^	2.30 ± 0.13 ^h^	1.41 ± 0.22	2.20 ± 0.24 ^h^	1.81 ± 0.19 ^h^	

Values found show means ± standard errors. Statistical analysis: Two-way ANOVA followed by Bonferroni test. Statistical difference between groups: a ≠ C (-); b ≠ C(+); c ≠ TAU (-); d ≠ (TAU (+); e ≠ 5FU (-), f ≠ 5FU (+), g ≠ TAU+5FU), h ≠ TAU +5FU (+); i ≠ all groups; *p* < 0.05.

**Table 3:** Percentages of adenomas and adenocarcinomas by group.

Group	Adenomas (%)	Adenocarcinomas (%)
C(+)	20%	80%
5FU(+)	30%	70%
TAU(+)	50%	50%
5FU+TAU(+)	100% adenomas	0% adenocarcinomas

**Table 4:** Results of immunohistochemical reactions for APC/β-catenin, p53, Ki-67, and K-ras.

Group	Control +	Taurine +	5-FU +	5-FU/Taurine +
	n = 20 (σ)	n = 13 (σ)	n = 20 (σ)	n = 12 (σ)
β-catenin (Q)	3.65 ± 0.67	3.92 ± 0.28	3.90 ± 0.31	4.00 ± 0.00
β-catenin (I)	2.40 ± 0.68	1.92 ± 0.86	3.00 ± 0.00	2.58 ± 0.79
β-catenin (S)	8.70 ± 2.87	7.62 ± 3.55	11.70 ± 0.92	10.33 ± 3.71 ^a,b^ ****^, c^ **
p-53 (Q)	0.30 ± 0.73	0.62 ± 1.12	0.50 ± 2.01	0.33 ± 0.49
p-53 (I)	0.20 ± 0.52	0.54 ± 0.78	0.10 ± 0.45	0.67 ± 0.98
p-53 (S)	0.40 ± 1.05	1.00 ± 2.20	0.10 ± 0.45	0.67 ± 0.98
K-ras (Q)	1.05 ± 1.50	1.31 ± 1.49	1.10 ± 1.48	1.92 ± 1.38
K-ras (I)	0.60 ± 0.88	0.77 ± 0.93	0.60 ± 0.75	1.42 ± 0.90
K-ras (S)	1.50 ± 2.33	1.69 ± 1.97	1.40 ± 2.06	2.92 ± 2.11 ^a,c^ *
Ki-67 (Q)	0.25 ± 0.44	0.46 ± 0.52	0.20 ± 0.41)	0.45 ± 0.52
Ki-67 (I)	0.80 ± 1.44	1.62 ± 1.56	0.75 ± 1.33	1.09 ± 1.30
Ki-67 (S)	0.80 ± 1.44	1.38 ± 1.56	0.60 ± 1.23	1.00 ± 1.28

(Q) = quantity; (I) = intensity; (S) = score; a = compared to control; b = compared to TAU; c = compared to 5-FU; * *p* < 0.5; ** *p* <0.01; **** *p* < 0.0001.

## Data Availability

The original contributions presented in the study are included in the article, further inquiries can be directed to the corresponding authors.
